# ﻿Two new species of *Itaphlebia* (Insecta, Mecoptera, Nannochoristidae) from the late Middle Jurassic of China

**DOI:** 10.3897/zookeys.1108.85378

**Published:** 2022-06-24

**Authors:** Yizi Cao, Xiaodan Lin, Chungkun Shih, Dong Ren

**Affiliations:** 1 College of Life Sciences, Capital Normal University, 105 Xisanhuanbeilu, Haidian District, Beijing 100048, China Capital Normal University Beijing China; 2 Key Laboratory of Green Prevention and Control of Tropical Plant Diseases and Pests, Ministry of Education, College of Plant Protection, Hainan University, Haikou, Hainan 570228, China Hainan University Haikou China; 3 Department of Paleobiology, National Museum of Natural History, Smithsonian Institution, Washington, DC, 20013–7012, USA National Museum of Natural History Washington, DC United States of America

**Keywords:** Insect fossil, Jiulongshan Formation, nannochoristid, taxonomy

## Abstract

Two new species of *Itaphlebia* Sukatsheva, 1985, *I.procera***sp. nov.** and *I.elegana***sp. nov.**, are described and illustrated from the latest Middle Jurassic Jiulongshan Formation of Daohugou, Inner Mongolia, China. Based on fossil specimens with wings, these new species are established and assigned to *Itaphlebia* by a combination of three forewing characters: Sc with three branches ending at C, the four-branched Rs (R_2_ to R_5_) originating distad of M (vs. three-branched RS (R_2+3_ undivided) in all other fossil and extant nannochoristids), and M forking with four branches; and a hind wing character of Sc simple and short, terminating at C well before the pterostigma. Furthermore, this is the first report of long and robust setae present on the anal veins of the forewing for *I.elegana***sp. nov.** in fossil Nannochoristidae.

## ﻿Introduction

Mecoptera Packard, 1886 is an order of insects, comprising nine extant families with more than 600 species (Soszyńska-Maj and Krzemiński 2013; Lin et al. 2016; Tillyard 1917). The earliest fossil records of mecopterans are known from the Early Permian, which occupy an important place in the Insecta. The insect fossil sites of Nannochoristidae Tillyard, 1917 are located in Siberia in Russia, Kazakhstan and Inner Mongolia, Liaoning in China. In the Southern Hemisphere, larval fossils from the Australian Cretaceous and eight extant species of nannochoristids have been documented in Argentina, Chile, New Zealand, Australia, and Tasmania (Jell and Duncan 1986; Byers 1989a; Ren et al. 2009; Cao et al. 2015). Based on conspicuously divergent larval morphology and lifestyle, Nannochoristidae has been suggested as a separate suborder, Nannomecoptera Hinton, 1981, to distinguish it from other families of Mecoptera (Penny 1975; Hinton 1981; Kristensen 1989). In recent phylogenetic studies, the systematic placement of Nannochoristidae has not been accurately settled. As a result of unusual and more primitive morphological characters of larval and immature stages (Beutel and Baum 2008; Beutel et al. 2009), Nannochoristidae together with Boreidae Latreille, 1816 form a clade with fleas, based more on phenetic differences than phylogenetic argumentation (Whiting 2002). Even though such thorough analyses have used extensive molecular data, the precise treatment and phylogenetic position of this separated suborder are still uncertain as well as unpredictable. Meanwhile, biological information on nannochoristids is limited; larvae are campodeiform, prognathous and predaceous, adults are almost aquatic, mostly resting or laying eggs on moist leaf litters at river edges. The adults may be omnivorous or nectarivorous, as documented studies on head morphology distinctly illustrate that they exclusively feed on flowing liquid, mainly nectar (Beutel and Baum 2008; Tillyard 1917; Beutel and Friedrich 2019). With their peculiar method of copulation and lacking detailed observations on feeding habits (Tillyard 1917), the lifestyle and biology of these rather fragile adults are still unclear so far.

Due to limited specimens of well-preserved compression fossils for nannochoristids, it was difficult to study detailed morphological characters to classify, diagnose and describe species or genera of these specimens. Therefore, the classifications of fossil species and genera of Nannochoristidae are in a state change and revision. So far, six genera: *Dahurochorista* Sukatsheva, 1985, *Dahurolarva* Sukatsheva, 1985, *Itaphlebia* Sukatsheva, 1985, *Namdyrus* Sukatsheva, 1993, *Tarantogus* Sukatsheva, 1985, and *Undisca* Sukatsheva, 1990, and one subgenus: *Eunannochorista* Novokshonov, 1997, from the Middle Jurassic and the Lower Cretaceous have been described (Sukatsheva 1985; Sukatsheva 1990; Novokshonov 1997a, b; Liu et al. 2010; Cao et al. 2015). However, the diagnoses of *Dahurolarva* and *Tarantogus* (Sukatsheva 1985) are based only on larvae, as revised by Novokshonov (1997b), and the diagnoses of *Undisca* and *Itaphlebia* (Sukatsheva 1985; Sukatsheva 1990) need revision after new and more well-preserved fossils have become available. In this paper, we describe two new species of *Itaphlebia*: *I.procera* sp. nov. and *I.elegana* sp. nov., and emend the diagnosis of *Itaphlebia*, based on five new and well-preserved fossil specimens.

All specimens were collected from the Jiulongshan Formation at Daohugou Village of Ningcheng County in Inner Mongolia. This locality is one of the richest Middle Jurassic fossil-bearing sites in China. The Jiulongshan Formation has yielded abundant and diverse mecopteran fossils (Cao et al. 2015), highlighting the complex interactions of mecopterans with the ecosystems (Wang et al. 2012). The deposits are considered as the latest Middle Jurassic (late Callovian) in age (Walker et al. 2013; Ren et al. 2019; Gao et al. 2021), approximately 165–164 Mya.

## ﻿Materials and methods

The new type specimens are collected from the latest Middle Jurassic, Jiulongshan Formation; Daohugou Village, Shantou Township, Ningcheng City, Inner Mongolia, China and housed in the insect fossil collection of the Key Laboratory of Insect Evolution and Environmental Changes, College of Life Sciences, Capital Normal University, Beijing, China (**CNUB**; Dong Ren, Curator).

These specimens were examined and photographed using a LEICA M165C dissecting microscope with a LEICA DFC 500 digital camera system with cool white transmitted light from double optical fibers, irradiating the specimens from two sides simultaneously, and illustrated with the aid of a drawing tube attachment. Enlarged photos were taken by using a Nikon SMZ 25 microscope with a Nikon DS-Ri 2 digital camera system. The line drawings were edited with Adobe Photoshop CS5. We use the venational nomenclature of Byers (1989b) as a frame of reference.

## ﻿Systematic palaeontology

**Order Mecoptera Packard, 1886**

**Family Nannochoristidae Tillyard, 1917**

### ﻿Genus Itaphlebia

Taxon classificationAnimaliaMecopteraNannochoristidae

Sukatsheva, 1985

CCE7BB9C-C408-5340-A58A-B29C53F40E22

Chrysopanorpa Ren, 1995, p. 91.
Netropanorpodes Sun, Ren & Shih, 2007, p. 867.
Stylopanorpodes Sun, Ren & Shih, 2007, p. 865.
Protochoristella Sun, Ren & Shih, 2007, p. 405.

#### Diagnosis.

Small to moderately-sized insect (forewing length 5.1–15.4 mm); body slender; wing nearly oval or rounded apically. Forewing: Sc with two or three branches ending at C; Rs (R_2_ to R_5_) origination distad of M origination; Rs forking with four (in some cases with five) branches; M forking with four (in some cases with five) branches. Hind wing: Sc simple and short, terminating at C well before pterostigma; Rs forking with four branches; M forking with four branches (in some cases with five).

#### Type species.

*Itaphlebiacompleta* Sukatsheva, 1985

#### Species included.

Type species, *I.ruderalis* comb. (= *Chrysopanorparuderalis* Ren, 1995 = *Stylopanorpodeseurypterus* Sun, Ren & Shih, 2007 = *Protochoristellaformosa* Sun, Ren & Shih, 2007), *I.jeniseica* Novokshonov, 1997 (= *Netropanorpodessentosus* Sun, Ren & Shih, 2007), *I.multa* Novokshonov, 1997 (= *Protochoristellapolyneura* Sun, Ren & Shih, 2007), *I.reducta* Novokshonov, 1997, *I.sharovi* Novokshonov, 1997, *I.generosa* Novokshonov & Sukatsheva, 2003, *I.decorosa* comb. (= *Netropanorpodesdecorosus* Sun, Ren & Shih, 2007), *I.exquisita* Liu, Zhao & Ren, 2010, *I.laeta* Liu, Zhao & Ren, 2010, *I.longiovata* Cao, Shih, Bashkuev & Ren, 2015, *I.amoena* Cao, Shih, Bashkuev & Ren, 2015, *I.procera* sp. nov. and *I.elegana* sp. nov.

### ﻿ Itaphlebia procera

Taxon classificationAnimaliaMecopteraNannochoristidae

Cao, Shih & Ren sp. nov.

7788B6F9-9FBF-5090-9E82-F05D7FD465B3

https://zoobank.org/3771FB50-DDB5-45CA-968A-8DC60CE923CF

Figs 1 , 2

#### Etymology.

The specific name is derived from the Latin adjective “procerus”, indicating the relatively large body size.

#### Type material.

**Figure 1.**
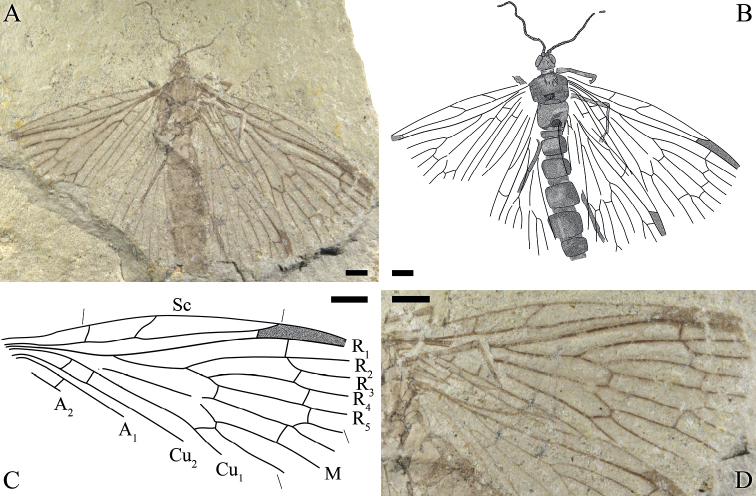
*Itaphlebiaprocera* sp. nov., holotype, CNU-MEC-NN2009273 **A** photograph of holotype **B** line drawing of holotype **C** line drawing of right forewing **D** photograph of right wing. Scale bars: 1 mm (**A–D**).

**Figure 2.**
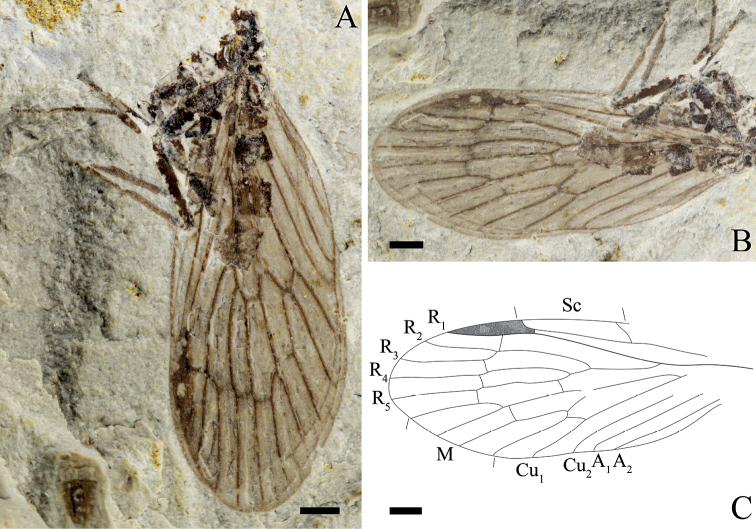
*Itaphlebiaprocera* sp. nov., paratype, CNU-MEC-NN2009168 **A** photograph of paratype **B** photograph of wings **C** line drawing of forewing. Scale bars: 1 mm (**A–C**).

***Holotype***: sex unknown, No. CNU-MEC-NN2009273 (Fig. 1), preserved in dorsal view, head and thorax well-preserved, with four wings partly preserved, veins of right forewing mostly discernible. ***Paratype***: sex unknown, No. CNU-MEC-NN2009168 (Fig. 2), preserved in lateral view, body partly preserved, only one forewing venation clearly discernible.

#### Locality and horizon.

All specimens were collected from the Jiulongshan Formation, latest Middle Jurassic age (Bathonian–Callovian boundary interval) from Daohugou Village, Ningcheng County, Inner Mongolia Autonomous Region in China.

#### Diagnosis.

Body size ca 10 mm. On forewing, Sc forking with three branches; R_2+3_ forking before M_1+2_; crossvein r_4_-r_5_ before crossvein r_5_-m_1_; Cu_1_+M forking before the crossvein cu_1_-cu_2_; and crossvein cu_1_-m_4_ after M_3+4_ forking.

#### Description.

Mainly based on Holotype, unless indicated as paratype.

***Head***: Oval with large compound eyes. Antenna partially preserved, filiform with 33 and 27 segments as preserved respectively. Vertex of the head raised, mouthparts long and slender.

***Thorax***: Long and relatively well-preserved, pronotum (width 0.5 mm, length 0.25 mm), mesonotum (width 1.5 mm, length 1.25 mm), metanotum (width 1.25 mm, length 1.4 mm) and scutellum (heavy shadowed) clearly discernible.

***Leg***: Leg barely preserved and slender, tibia longer than femur, surfaces of all legs densely covered with short and irregularly arranged setae.

***Wing***: Forewing: Long and oval, basal part of the wing narrow, gradually broadening from the base toward the apex. Right forewing with the anterior area broad, a distinct pterostigma present; Sc with 3 branches, Sc_1_ and Sc_2_ ending at C before the middle of the wing length, Sc_3_ terminated at C right near pterostigmal area; R forking before Sc_2_, Rs forking with 4 branches, one short crossvein sc-r before pterostigma, crossvein r_1_-r_2_ under the pterostigma, approximately near the forking of R_2+3_, crossvein r_3_-r_4_ after the level of the crossvein r_1_-r_2_, R_2+3_ 4 times as long as R_4+5_, crossvein r_5_-m_1+2_ emerged after the forking of R_4+5_, oblique crossvein r_5_-m_1_ present after the crossvein r_4_-r_5_; conspicuous thyridium at the forking of M; M with 4 branches, M_3+4_ divided beyond the forking of M_1+2_; straight crossvein m_1+2_-m_3_ between M_1+2_ and M_3_, near the forking of M_1+2_; crossvein m-cu_1_ after the forking of M_3+4_, somewhat S-shaped; Cu_1_ coalesced with M for a long distance and separated from M after the crossvein cu_1_-cu_2_; A_1_ and A_2_ are almost parallel; crossvein a_1_-a_2_ after the level as the base of Cu_1_; A_3_ absent. Hind wing: Shorter than forewing; the pterostigma slightly darkened. Sc simple, terminated at C in the first one third of wing, one crossvein c-r_1_ present at half-length of hind wing; R_1_ under pterostigma and connected with C by a short crossvein c-r_1_; posterior and anal margins in hind wings not preserved.

***Abdomen***: With six visible large abdominal segments, segments II–IV and segment VI distinctly smaller than segment V, the distal segments not preserved.

#### Remarks.

The new species shows great differences from other species of *Itaphlebia* in having relatively moderate body size, a broader thoracic notum and the presence and position of the crossvein r_5_-m_1_ that is in a more distal position than in other species, which is also a new finding for nannochoristid venation. So far, we have found two specimens of the new species, of which characters appear to be stable and therefore sufficient for establishing a new species.

#### Measurements

**(in mm). *Holotype***: No. CNU-MEC-NN2009273: head length 0.7 (excluding the antenna), head maximum width 1.0 (excluding the antenna), thorax length ca 3.0, thorax maximum width 1.9, forewing length 9.4, hind wing length 7.5 (all as preserved). ***Paratype***: No. CNU-MEC-NN2009168: forewing length 11.5, forewing maximum width 4.4 (all as preserved).

### ﻿ Itaphlebia elegana

Taxon classificationAnimaliaMecopteraNannochoristidae

Cao, Shih & Ren sp. nov.

FFD78591-41C3-5252-807D-8FA4EB7B719C

https://zoobank.org/3D8414C6-F617-4F7B-BC84-13330D7A5A5D

Figs 3 , 4 , 5

#### Etymology.

The specific name is from Latin adjective “elegans”, referring to the elegant body posture.

#### Type material.

**Figure 3.**
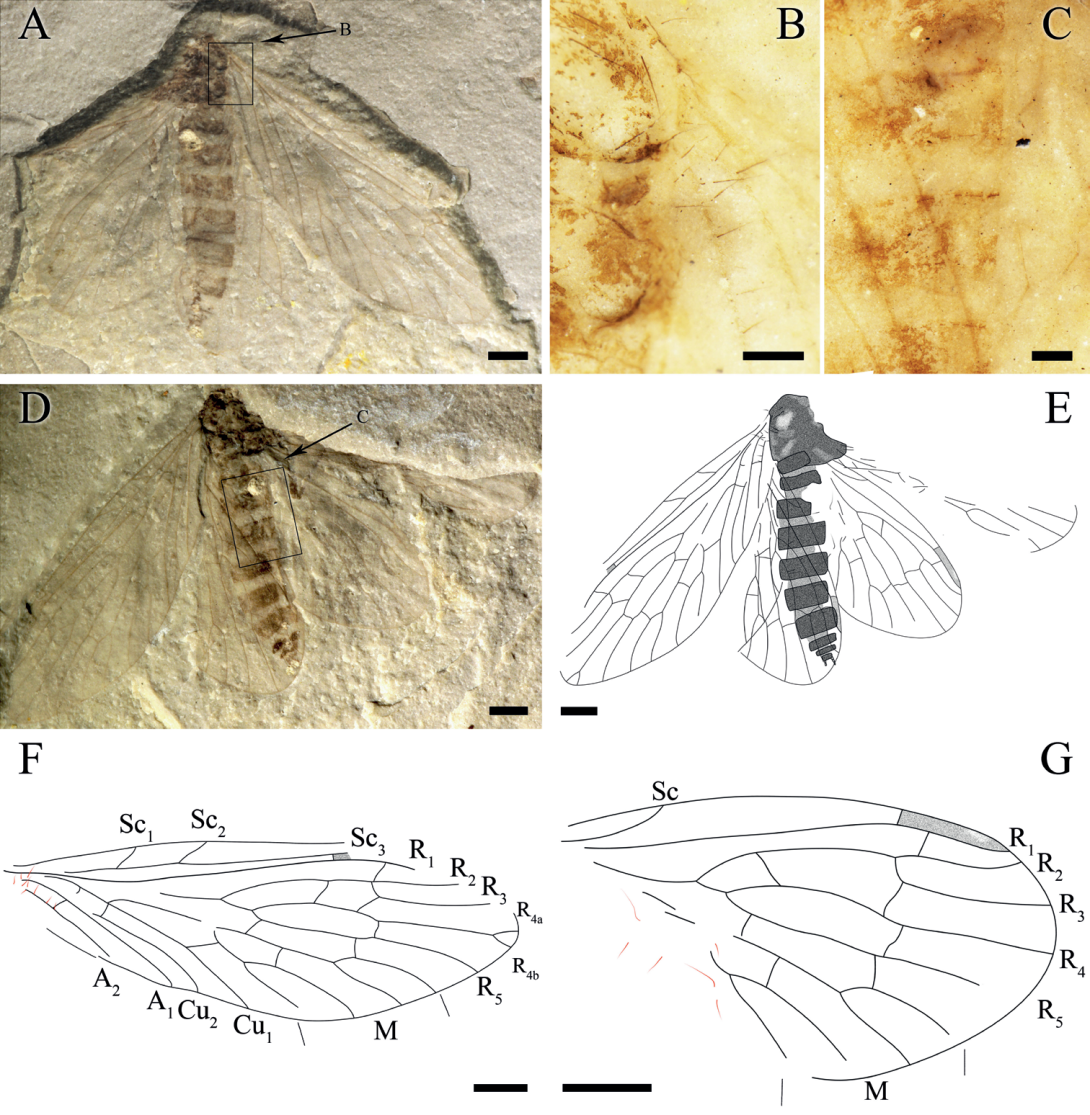
*Itaphlebiaelegana* sp. nov., holotype, CNU-MEC-NN2008228 p/c. **A** photograph of part **B** enlarged details of basal forewing of part, anal veins with long and robust setae (under alcohol) **C** enlarged details of basal hind wing of counterpart (under alcohol) **D** photograph of counterpart **E** line drawing of counterpart **F** Line drawing of forewing of part, anal veins with long and robust setae **G** line drawing of hind wing of counterpart. Scale bars: 1 mm (**A–G**).

**Figure 4.**
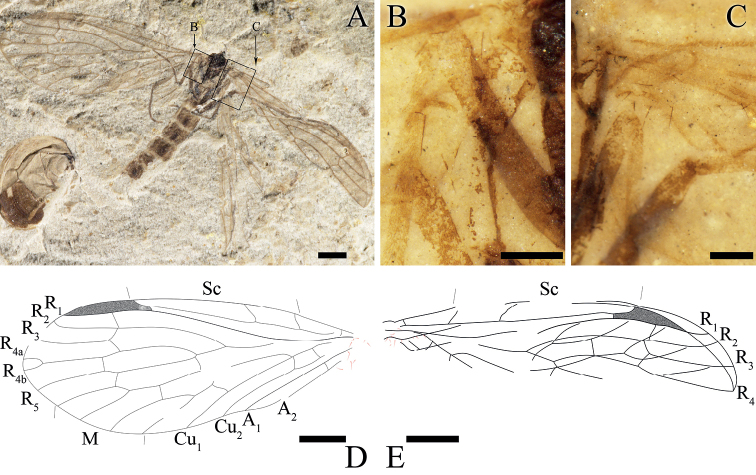
*Itaphlebiaelegana* sp. nov., paratype, CNU-MEC-NN2015026 **A** photograph of paratype **B** enlarged details of base of right forewing (under alcohol) **C** enlarged details of base of left forewing (under alcohol) **D** line drawing of right forewing **E** line drawing of left forewing. Scale bars: 1 mm (**A–E**).

**Figure 5.**
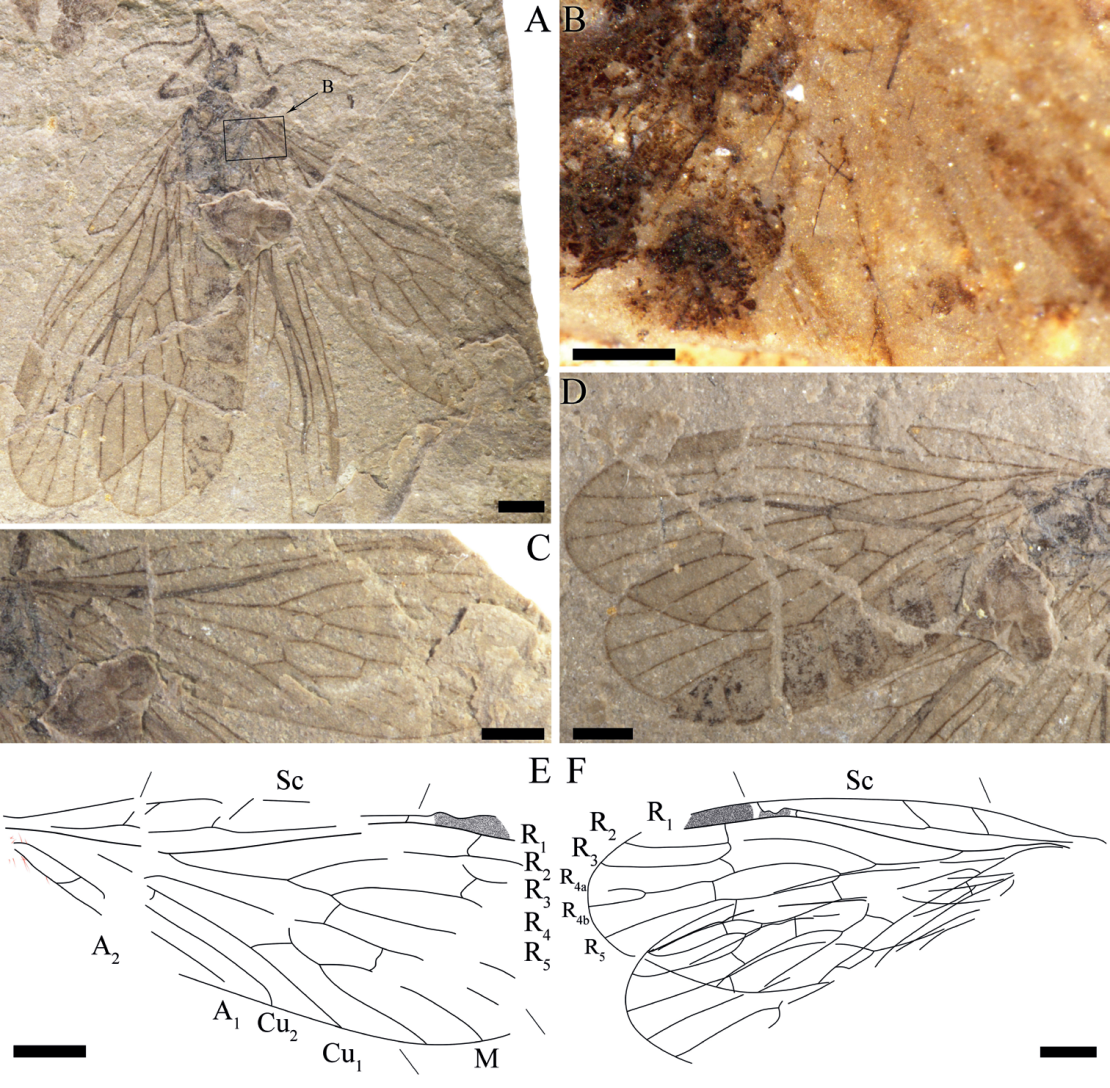
*Itaphlebiaelegana* sp. nov., paratype, CNU-MEC-NN2016020 p/c. **A** photograph of part of paratype **B** enlarged details of base of forewing of counterpart of paratype (under alcohol) **C** photograph of right forewing of part **D** photograph of left forewing and part of hind wing of part **E** line drawing of right forewing **F** line drawing of left forewing and part of hind wing. Scale bars: 1 mm (**A–F**).

***Holotype***: female, No. CNU-MEC-NN2008228 p/c (Fig. 3), preserved in lateral view, thorax partly preserved, abdomen well-preserved, with four wings partly preserved, veins of one forewing mostly discernible, veins of one hind wing relatively discernible. ***Paratypes***: sex unknown, No. CNU-MEC-NN2015026 (Fig. 4), preserved in ventral view, thorax and abdomen partly preserved, only right forewing venation clearly discernible; female, CNU-MEC-NN2016020 p/c (Fig. 5), body nearly complete but not well-preserved, forewing venation partly discernible.

#### Locality and horizon.

All specimens were collected from the Jiulongshan Formation, latest Middle Jurassic age (Bathonian–Callovian boundary interval) from Daohugou Village, Ningcheng County, Inner Mongolia Autonomous Region in China.

#### Diagnosis.

On forewing, costal area slightly broad; Sc forking with three branches; Rs (R_2_ to R_5_) forking with five branches; R_2+3_ forking before the crossvein r_3_-r_4_; R_4+5_ forking before the crossvein r_5_-m_1+2_; crossvein cu_1_-cu_2_ near the forking of Cu_1_+M, and crossvein cu_1_-m_4_ far before M_3+4_ forking. Long and robust setae present on anal veins of forewing.

#### Description.

Mainly based on Holotype, unless indicated as paratype.

***Thorax***: Incompletely preserved, setae discernible.

***Wing***: Long and oval, basal part of the wing narrow, gradually broadening from the base toward the apex. Forewing with the basal part of C somewhat convex, anterior area slightly broad, pterostigma incompletely preserved; Sc with three branches, Sc_1_ and Sc_2_ ending at C before the middle of the wing length, Sc_3_ near pterostigmal area; Rs forking with five branches, one short crossvein sc-r before pterostigma, crossvein r_1_-r_2_ under the pterostigma, approximately near the forking of R_2+3_, crossvein r_2+3_-r_4_ before crossvein sc-r, oblique crossvein r_3_-r_4_ between R_3_ and R_4_, R_2+3_ four times as long as R_4+5_, R_4_ forking with two branches (R_4a_, R_4b_), oblique crossvein r_5_-m_1+2_ emerged after the forking of R_4+5_, crossvein r_5_-m_1_ present before the crossvein r_4_-r_5_; conspicuous thyridium at the forking of M; M with four branches, M_3+4_ divided beyond the forking of M_1+2_; straight crossvein m_1+2_-m_3_ forking at the middle length of M_1+2_; crossvein m-cu_1_ before the forking of M_3+4_; Cu_1_ coalesced with M for a relatively short distance and separated from M near the crossvein cu_1_-cu_2_; long and robust setae present on veins A_1_ and A_2_, A_1_ and A_2_ almost parallel; crossvein a_1_-a_2_ at the same level of Cu_1_; A_3_ absent. Hind wing shorter and broader than forewing; pterostigma preserved in hind wing. Sc simple, terminated at C in the first one third of wing; R_1_ under pterostigma and connecting with C by a short crossvein c-r_1_, Rs with four branches, two crossveins between R_2+3_ and R_4_. Anal margins in hind wings not preserved, several long and robust setae preserved.

***Abdomen***: With 11 visible abdominal segments, segments VIII–XI distinctly smaller than segment VII, a pair of cerci at the end of abdomen, each one with three segments, setae discernible.

#### Remarks.

*Itaphlebiaelegana* sp. nov. demonstrates an individual aberration of the sixth branch of R vein by the forking of R_4_, providing a character for understanding the evolution of wing venation. Although the hind wing has R with five branches, the diagnosis of *Itaphlebia* should be revised to indicate that the forewing Rs forking with four or five branches. CNU-MEC-NN2008228 p/c has two crossveins between R_1+2_ and R_3+4_, but only one crossvein is visible in CNU-MEC-NN2016020 p/c and CNU-MEC-NN2016026 p/c. Considering these three specimens have many similarities regarding other characters, we regard them as the same species. The difference in crossveins between R_2+3_ and R_4_ might have been due to the intraspecific difference or inheritable mutations.

#### Measurements

**(in mm). *Holotype***: female, No. CNU-MEC-NN2008228 p/c: Abdomen length 6.0, forewing length 9.4, hind wing length 7.5 (all as preserved). ***Paratypes***: sex unknown, No. CNU-MEC-NN2015026: thorax length 1.8, thorax maximum width 1.1, forewing length 7.5, forewing maximum width 3.0 (all as preserved); female, CNU-MEC-NN2016020 p/c: head length 1.1 (excluding the antenna), head maximum width 0.8 (excluding the antenna), thorax length 2.2, thorax maximum width 1.7, forewing length 8.4, forewing maximum width 3.5 (all as preserved).

## ﻿Discussion

Taxonomic and morphological characters for fossil nannochoristids from the latest Middle Jurassic Jiulongshan Formation in Northeastern China have been investigated. *Itaphlebiaelegana* sp. nov. is unique in having a forewing with R_4_ forking with two branches and remarkable long setae on the anal veins. Novokshonov (1997a) thoroughly studied the difference in venation [mainly in Sc branches of forewing and Rs (R_2+3_) branches of both wings] between Mesozoic nannochoristids and the only extant genus, *Nannochorista* including, *N.dipteroides* Tillyard, 1917 (Tasmania); *N.eboraca* Tillyard, 1917 (New South Wales); *N.holostigma* Tillyard, 1917 (Tasmania); *N.maculipennis* Tillyard, 1917 (Tasmania); *N.neotropica* Navás, 1928 (western Argentina, Neuquén, Chile, Arauco, LLanquihue, Chiloe, Magellanes); *N.edwardsi* Kimmins, 1929 (western Argentina, Chile); *N.andina* Byers, 1989 (western Argentina, Neuquén); *N.philpotti* (= *Choristellaphilpotti* Tillyard, 1917, = *Microchoristaphilpotti* Byers, 1974) Kristensen, 1989 (New Zealand). In fossil nannochoristids, Sc has three branches (most species) on the forewing, terminating at C and Rs has four branches (five branches only in *I.elegana* sp. nov.), while all extant species have Sc with two branches, Sc_2_ fused with R_1_ for a distance (most species) on the forewing; and Rs with three branches. Therefore, the wing venation in Nannochoristidae has become simplified over time. On the other hand, the distance from the forking point of R_4a+b_ to the margin of the forewing is 0.5 mm for the holotype of *I.elegana* sp. nov. (vs. 0.5 mm and 1.1 mm for the paratypes), indicating the occurrence of such a forking of R_4a_ and R_4b_ in the forewing is frequent. Hence, the forking of R_4_ should be a character for *I.elegana* sp. nov. and the diagnosis of *Itaphlebia* is also emended.

*Itaphlebiaelegana* sp. nov. has distinct and short setae on the anal veins of the forewing, which was described in only one extant species of Nannochoristidae, *Nannochoristaandina* (Byers 1989a). The setae of *I.elegana* sp. nov. are similar in appearance to those of the two fossil species, *Jurassipanorpasticta* Ding Shih & Ren, 2014 and *Jurassipanorpaimpunctata* Ding Shih & Ren, 2014 in Panorpidae Latreille, 1805 (Ding et al. 2014) and the extant *Notiothaumareedi* in Eomeropidae Cockerell, 1909 (Cockerell 1909; Crampton 1930; Mickoleit 1971). The distinct and short setae on forewing anal veins of *I.elegana* sp. nov. are different from microtrichia on the wing membrane or the dense and shorter setae of extant species of Panorpidae as well as the notably long and robust setae for the extinct species of Orthophlebiidae Handlirsch, 1906 (Mickoleit 1971; Ding et al. 2014).

Furthermore, such setae in *Itaphlebiaelegana* sp. nov. are apparently shorter and less powerful, compared to the many stout bristles (dinotrichia) on the basal anterior margin of the forewings of fossil eomeropids of *Tsuchingothaumashihi* Ren & Shih 2005 from the latest Middle Jurassic and *Typhothaumayixianensis* Ren & Shih 2005 from the Early Cretaceous, both in Northeastern China (Ren and Shih 2005), and of both fore- and hind wings of the extant *N.reedi* (Carpenter 1909, Crampton 1930; Mickoleit 1971; Carpenter 1972). But such setae in *I.elegana* sp. nov. might be homologous to those of *N.reedi* as they all attach to the basal parts of the wings (Carpenter 1972; Crampton 1930; Mickoleit 1971). The setae of *N.reedi* are located on the middle or anal parts of the forewing, gradually getting smaller and only emerging on the basal region of some veins in the forewing. Such long bristles in the hind wing are usually the forerunners of the frenulum of higher insects on the front edge of the hind wing, which is hardly called a frenulum on the forewing (Crampton 1930). Therefore, the functions of these setae on forewings remain puzzling. Due to the setae occurring only on the anal veins of the forewings for *I.elegana* sp. nov., we surmised that they might have been sensory or used for wing coupling (Byers 1989a; Ding et al. 2014). However, since we have not found any other associated structures preserved on the anterior part of the hind wings of these fossil specimens as well as extant species, the functions of these setae are still unknown.

Compared with other species of *Itaphlebia*, *I.procera* sp. nov. has unique venational characters: crossvein r_5_-m_1_ distad of the levels of crossveins between R_1_ and Rs, namely the forking of M_1+2_ originates basal (for Fig. 2C) or distad (for Fig. 1C) of the forking of R_2+3_; and crossvein m_1+2_-m_3_ is near the level of the crossvein between R_1_ and Rs (vs. basal location in most species). Hence, we propose that the origin of M_1+2_ is variable in *Itaphlebia*, and it should be a diagnosis at the level of species for *Itaphlebia*.

## ﻿Conclusions

Eight extant species of Nannochoristidae Tillyard, 1917 have been documented in the Southern Hemisphere. The fossil sites of nannochoristids are in Siberia in Russia, Kazakhstan and Inner Mongolia and Liaoning in China. Due to limited specimens of well-preserved compression fossils, it was difficult to study detailed morphological characters to classify, diagnose and describe species or genera of these fossil nannochoristids.

We describe two new species of an extinct genus, *Itaphlebiaprocera* sp. nov. and *I.elegana* sp. nov., in the family Nannochoristidae, based on five fossil specimens from the latest Middle Jurassic Jiulongshan Formation of Daohugou, Inner Mongolia, in Northeastern China. *Itaphlebiaelegana* sp. nov. demonstrates a new and unique forking of R_4_ into two branches (R_4a_, R_4b_), providing a character for understanding the evolution of wing venation. Furthermore, we report long and robust setae present on the anal veins of the forewing in *I.elegana* sp. nov. It is the first time that these two characters of *Itaphlebia* are documented for the fossil records in Nannochoristidae.

These new species not only broadened the diversity of the *Itaphlebia* in the mid-Mesozoic ecosystems but also provided new taxonomic information to emend the generic diagnosis.

## Supplementary Material

XML Treatment for Itaphlebia

XML Treatment for Itaphlebia procera

XML Treatment for Itaphlebia elegana
